# The dynamic changes of HBV quasispecies diversity in infancy after immunoprophylaxis failure: a prospective cohort study

**DOI:** 10.1186/s12985-021-01707-9

**Published:** 2021-11-29

**Authors:** Yi Li, Yiwei Xiao, Lili Li, Yarong Song, Xiangjun Zhai, Jianxun Liu, Zhongping Duan, Ling Yan, Feng Ding, Jia Liu, Liguo Zhu, Jie Jiang, Huaibin Zou, Lingxiang Li, Caihong Liang, Jie Wang, Jie Li

**Affiliations:** 1 Department of Microbiology and Infectious Disease Center, School of Basic Medical Sciences, Peking University Health Science Center, 38 Xueyuan Road, Haidian District, Beijing, 100083 China; 2 Jiangsu Provincial Center for Disease Control and Prevention, Nanjing, 210009 China; 3 Zhengzhou Municipal Center for Disease Control and Prevention, Zhengzhou, 450053 China; 4 Beijing Youan Hospital, Capital Medical University, Beijing, 100054 China; 5 Gongyi City Maternal and Child Health Hospital, Zhengzhou, 451200 China; 6 Zhongmu County Maternal and Child Health Hospital, Zhengzhou, 451450 China

**Keywords:** Hepatitis B virus, Quasispecies, Mother-to-child transmission, Immunoprophylaxis failure, Infantile antiviral therapy

## Abstract

**Background:**

Previous works have observed that younger infants with chronic hepatitis B virus (HBV) infection are more responsive to antiviral treatment. However, the underlying mechanism remains unclear. In this study, the dynamic changes of HBV quasispecies in infants with immunoprophylaxis failure were investigated to provide virological explanations for clinical management on infantile antiviral therapy.

**Methods:**

Thirteen 7-month-old infants with immunoprophylaxis failure and their mothers were enrolled from a prospective cohort, and 8 of them were followed up to 3 years old. The sequences of HBV quasispecies were analyzed by the full-length genome clone-based sequencing, and compared among mothers and their infants at different ages.

**Results:**

The results revealed that the complexity, mutation frequency and genetic distance of HBV quasispecies decreased significantly at full-length, partial open reading frames and regulatory regions of HBV genome at nucleotide level in 7-month-old infants comparing with their mothers, whereas increased significantly to near the maternal level when infants grew up to 3 years old. Furthermore, similar changes were also found in Core, PreS2, RT and P regions of HBV genome at amino acid level, especially for potential NAs-resistant mutants in RT region and immune-escape mutants in Core and PreS2 regions.

**Conclusions:**

This study uncovered the evolution of HBV quasispecies in infancy after mother-to-child transmission, which may provide the virological evidence for explaning that younger children are more responsive to antiviral therapy.

**Supplementary Information:**

The online version contains supplementary material available at 10.1186/s12985-021-01707-9.

## Introduction

Mother-to-child transmission (MTCT) remains one of the predominant routes of hepatitis B virus (HBV) dissemination worldwide. Infants with chronic HBV infection may suffer impaired quality of life and have a higher lifetime risk of developing end-stage liver disease than adults [1]. It has been shown that the risk of hepatocellular carcinoma (HCC) is higher in Asian population with prenatally acquired HBV as compared with horizontally acquired HBV [2]. Therefore, chronicity comes as a serious threat to HBV-infected infants, and appropriate clinical managements for them are in urgent need.

Fortunately, most of the studies conducted on antiviral therapy in pediatric chronic hepatitis B (CHB) patients have shown promising results with significant improvements in the rates of viral control, HBeAg seroconversion and HBsAg loss [3–9]. Meanwhile, the antiviral efficacy of HBV-infected infants at immune tolerant (IT) stage also showed encouraging outcomes with 61–78% of infants achieved HBV DNA loss, 22–39% achieved HBeAg seroconversion, and 17–22% achieved HBsAg loss [10–13]. Moreover, a recent study showed that the infants receiving antiviral therapies before 1 year old obtained more benefits than those receiving antiviral therapies after 1 year old, with 83% of HBsAg loss, shorter treatment duration and lower incidence of adverse events [14]. Overall, most of the studies showed that the younger patients were more responsive to antiviral treatment, suggesting the timing of the initial treatment is crucial. However, the underlying mechanisms are unclear.

Interestingly, several studies revealed that antiviral treatment responders generally had a lower quasispecies complexity than the non-responders [15–18]. Besides that, our previous study [19] revealed a significant lower HBV quasispecies diversity in mothers of infants with immunoprophylaxis failure. However, the HBV quasispecies characteristics of these infants prenatally infected are unclear, and its correlation with antiviral efficacy are unknown. Especially, the dynamic changes of HBV quasispecies during MTCT and evolution in the early stage of infection have not been well defined.

In this study, both 7-month-old infants with immunoprophylaxis failure and their mothers were included, and some infants were followed up to 3 years old. The dynamic changes of HBV quasispecies characteristics were analyzed by the full-length HBV genome clone-based sequencing assay among mothers and their paired infants. This study aims to explore the evolution of HBV quasispecies in infants with immunoprophylaxis failure, which may provide a virological evidence for establishing the optimal approach to the clinical management of infants with chronic HBV infection.

## Material and methods

### Subjects

As previously reported in our prospective mother-infant paired study [20], total 1177 infants were returned for post-vaccination serologic testing at 7 months, and 20 of 1177 infants were immunoprophylaxis failure after the full course of vaccination. Among 20 infants with immunoprophylaxis failure, 15 infants were infected with genotype C HBV. The other 3 infants infected with genotype B HBV and 2 infants who were not able to identify genotype due to insufficient sera were excluded to avoid the potential impact of genotype on sequence mutation analysis. Therefore, 15 mother-infant pairs infected with genotype C HBV were enrolled in this study, whereas the full-length HBV genome clones were successfully obtained from 13 infants. The blood samples of mothers were collected before delivery. Eight of the 13 infants were followed up to 3 years old. All mothers were antiviral-naïve, and infants were received 0-1-6 vaccination program, combined with one dosage of HBIG within 12 h of birth.

### Amplification, cloning and sequencing

HBV genomes were extracted from 200 µL serum samples using QIAamp DNA blood mini kit (Qiagen, Hilden, Germany). The HBV genome was amplified by PCR as described by previous study [19], and followed by clone-based sequencing. Details and primers are provided in Additional file 1. PCR products of about 3200 bp were purified and cloned into the pGEM^®^-T Easy Vector Systems (Promega, Beijing, China) after the addition of adenylate tails.

### Sequence analyses

Sequence segments were assembled to full-length HBV genome and divided into 11 coding and 7 noncoding regions. Details and nucleotide sites of these regions were provided in Additional file 1. As reported by our previous study [19], viral quasispecies characterization was evaluated by three parameters at both nucleotide and amino acid level: mutation frequency, complexity (Shannon entropy) and diversity (mean genetic distance). Quasispecies complexity was measured using normalized Shannon entropy (Sn) [Sn =  − Σi (pi × lnpi)/lnN] where N is the total number of clones, and pi is the percentage of each clone in the viral quasispecies population. Genetic distance was calculated at the nucleotide level under Tamura 3-parameter method and at the amino acid level under Jones-Taylor-Thornton (JTT) matrix-based method. The number of synonymous substitutions per synonymous site (dS) and the number of nonsynonymous substitutions per nonsynonymous site (dN) were calculated under modified Nei-Gojobori model with Jukes-Cantor correction. The phylogenetic analyses were performed by neighbor-joining tree constructed by Tamura 3-parameter model given in the MEGAX software. Mutations were identified by a same consensus sequence synthesized by all clones from mothers. B cell and CD4+ T cell immune epitopes in Core and PreS2 regions were identified based on previous publications [21–23].

### Serological assays

Serum HBsAg and HBeAg were tested by Abbott chemiluminescent microparticle immunoassay (Abbott Diagnostic, Chicago, IL, USA), as well as HBV DNA load was measured by Abbott real-time HBV DNA assay (Abbott Molecular, IL, USA).

### Statistical analyses

Categorical variables were expressed as % (m/n) and examined by χ^2^/Fisher’s exact test. Non-normal distributions data were expressed as median and IQR or (range) and compared by Wilcoxon Signed Rank Test. All *P* values were two-tailed and a *P* value < 0.05 was considered significant. Statistical analyses were analyzed using SPSS software V.25.0 (Chicago, IL, USA).

## Results

### Demographic and virological data

Totally 13 7-month-old infants and their mothers were enrolled, and 8 of them were followed up to 3 years old. The levels of serum HBV DNA, HBsAg and HBeAg did not change significantly among mothers, 7-month-old infants and 3-year-old infants (Table 1). In addition, an average of 16 ± 4.72 full-length HBV genome clones per sample were collected, and no significant differences of the clone numbers were noticed among groups.

**Table 1:** The demographic, clinical and laboratory data of 13 mother-infant pairs

Subject		Age	Gender	Clone number	HBsAg (log_10_IU/ml)	HBeAg (log_10_S/CO)	HBV DNA (log_10_IU/mL)
Pair 1	M1	19Y		17	4.41	3.16	8.40
	C1-1	7M	Male	24	4.01	3.00	7.65
	C1-2	3Y		25	4.05	3.07	7.87
Pair 2	M2	21Y		19	4.52	3.04	8.23
	C2-1	7M	Male	24	2.19	2.12	5.87
	C2-2	3Y		19	3.60	3.15	7.80
Pair 3	M3	19Y		13	4.46	3.15	8.47
	C3-1	7M	Male	22	4.52	3.09	8.39
	C3-2	3Y		17	4.54	3.08	8.48
Pair 4	M4	32Y		19	4.83	3.18	8.24
	C4-1	7M	Female	10	5.14	3.24	7.97
	C4-2	3Y		20	5.01	3.18	8.16
Pair 5	M5	27Y		12	4.81	2.93	9.22
	C5-1	7M	Male	20	4.61	2.95	9.13
	C5-2	3Y		18	4.88	2.25	8.26
Pair 6	M6	23Y		9	4.38	3.21	8.12
	C6-1	7M	Female	8	4.58	3.03	8.87
	C6-2	3Y		13	4.69	3.19	8.06
Pair 7	M7	22Y		15	4.55	3.23	8.49
	C7-1	7M	Female	12	4.96	3.07	9.37
	C7-2	3Y		12	5.07	3.07	8.89
Pair 8	M8	20Y		14	4.43	3.11	8.08
	C8-1	7M	Male	13	4.58	3.19	8.97
	C8-2	3Y		17	4.09	3.07	8.39
Pair 9	M9	20Y		14	4.69	3.12	8.41
	C9	7M	Female	18	4.74	3.77	8.81
Pair 10	M10	29Y		14	4.25	2.90	8.82
	C10	7M	Female	19	4.92	2.93	8.16
Pair 11	M11	22Y		10	4.73	2.94	8.96
	C11	7M	Female	22	4.19	0.85	8.67
Pair 12	M12	25Y		17	4.49	2.99	8.22
	C12	7M	Male	20	4.70	2.94	8.87
Pair 13	M13	20Y		7	3.92	3.16	7.82
	C13	7M	Male	14	3.79	3.24	8.32

M1: Mother 1; C1-1: child of mother 1 at 7 months; C1-2: child of mother 1 at 3 years; Y: years; M: months

The phylogenetic trees were constructed between 13 mothers and their paired 7-month-old infants, 8 infants at 7 months and 3 years old, as well as 8 mothers and their paired infants at 3 years old (Additional file 1: Figure S1). All clones clustered together with the reference sequence of genotype C HBV, and the sequences of all clones from the same pair were clustered together, indicating there was no contamination during the acquisition of clones.

### Comparative analysis of HBV quasispecies characteristics in mothers and their paired 7-month-old infants

**Fig. 1:**
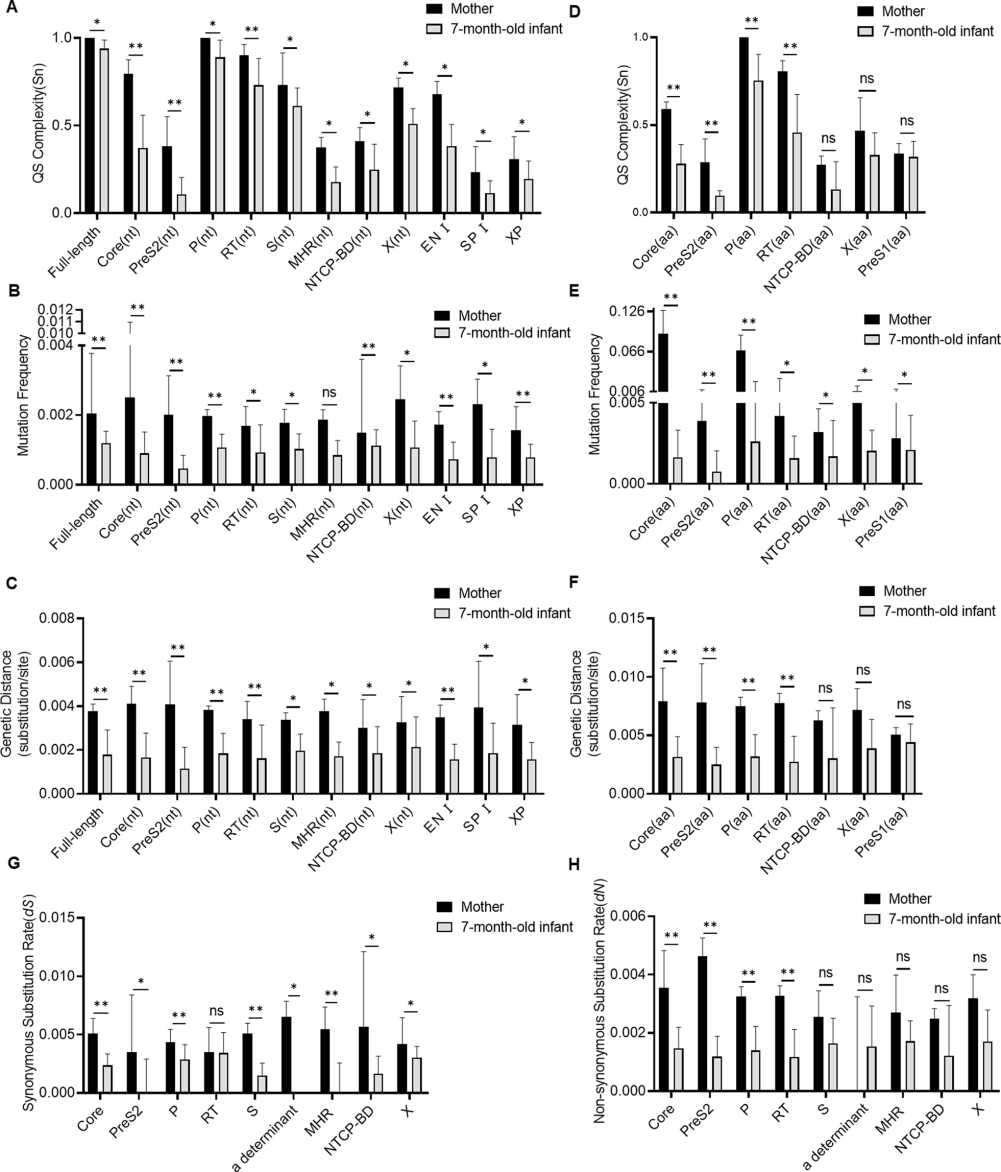
Comparative analysis of HBV quasispecies characteristics during MTCT. The quasispecies complexity, mutation frequency and genetic distance were analyzed between mothers and their paired 7-month-old infants at nucleotide level (**A**, **B** and **C**) and amino acid level (**D**, **E** and **F**), respectively. The comparison of synonymous substitution rate (*dS*) (**G**) and non-synonymous substitution rate (*dN*) (**H**). *represents *P* < 0.05. **represents *P* < 0.01. “nt” represents the nucleotide level. “aa” represents the amino acid level. NTCP-BD: sodium taurocholate cotransporting polypetide binding domain. MHR: major hydrophilic region. Quasispecies complexity was measured using normalized Shannon entropy (Sn). Genetic distance was calculated at the nucleotide level under Tamura 3-parameter method and at the amino acid level under Jones–Taylor–Thornton matrix-based method. dS and dN were calculated under modified Nei–Gojobori model with Jukes–Cantor correction

The quasispecies characteristic values, including complexity, mutation frequency and genetic distance, at full-length genome level, 11 coding regions and 7 noncoding regions were analyzed between mothers and 7-month-old infants. As shown in Additional file 1: Table S1 and Fig. 1A–C, the complexity, mutation frequencies and genetic distances of full-length genome and most specific regions at nucleotide level in 7-month-old infants were significantly lower than those in mothers.

For coding regions, the mutation frequencies of Core, PreS2, RT, NTCP-BD, X and PreS1 regions, as well as the complexities and genetic distances of Core, PreS2, P and RT regions at amino acid level in 7-month-old infants were also significantly lower than those in mothers (Additional file 1: Table S2 and Figs. 1D–F). Further, the synonymous substitution rates of most regions and the non-synonymous substitution rates of Core, PreS2, P and RT regions were dropped significantly after MTCT (Additional file 1: Table S3 and Fig. 1G, H).

The phylogenetic tree and mutation rate of single nucleotide site analyses were performed on each pair of infants and their mothers. Phylogenetic trees of HBV sequences in 10 infants (76.92%, 10/13) segregated from those of mothers, indicating the evolve selection was present during MTCT of HBV (Additional file 1: Figure S2). The mutation rates of some nucleotide sites were significantly different in 10 pairs of infants and their mothers (Table 2). In one of the other 3 pairs with sequences mixed in phylogenetic trees, C1826T and A1827C mutations were present in 40% of HBV sequences in infant, which is significantly higher than that of mother (Table 2).

**Table 2:** Detailed nucleotide site with mutation rate changed significantly during MTCT

Case	Mutants	MR (mother)	MR (7-month-old infants)	MR change	*P*	Amino acid substitution
Pair 1	G375T	0.06	0.38	0.32	0.028	sW74L
	C2102T	0.12	0.46	0.34	0.039	non
Pair 2	C339A	0.00	0.58	0.58	< 0.001	sP62L
	T2555C	0.00	0.42	0.42	0.001	non
	A2590T	0.00	0.42	0.42	0.001	pY95F
Pair 3	C105T	0.00	1.00	1.00	< 0.001	PreS2A39V
Pair 4	C1826T&A1827C	0.00	0.40	0.40	0.009	PrecH5S;xT152P
Pair 5	C2366A	0.00	0.50	0.50	0.004	cP156T
Pair 6	T3116C	0.00	0.94	0.94	< 0.001	PreS1V90A
Pair 7	T2708G	0.00	0.95	0.95	< 0.001	non
Pair 8	G648C	0.00	1.00	1.00	< 0.001	sW165S
	C732T	0.00	0.38	0.38	0.016	sS193L
Pair 9	C1T	0.50	0.00	− 0.50	0.001	non
	G20A	0.00	1.00	1.00	< 0.001	PreS2A11T
	A616G	0.43	0.00	− 0.43	0.003	rtI163V
	T999A	0.36	1.00	0.64	< 0.001	non
	C1913A	0.50	0.00	− 0.50	0.001	cP5T
	A2159G	0.50	0.00	− 0.50	0.001	cS87G
	A2189C	0.50	0.00	− 0.50	0.001	cI97L
Pair 10	C26A	0.29	0.00	− 0.29	0.024	PreS2L13I
	T39A	0.00	0.95	0.95	< 0.001	PreS2V17E
	G1386A	0.50	1.00	0.50	0.001	xV5M
	C2660T	0.29	1.00	0.71	< 0.001	non
Pair 11	T2576C	0.00	1.00	1.00	< 0.001	non

MR: Mutation rate at single nucleotide site

### Dynamics of HBV quasispecies characteristics from 7 months to 3 years old

**Fig. 2:**
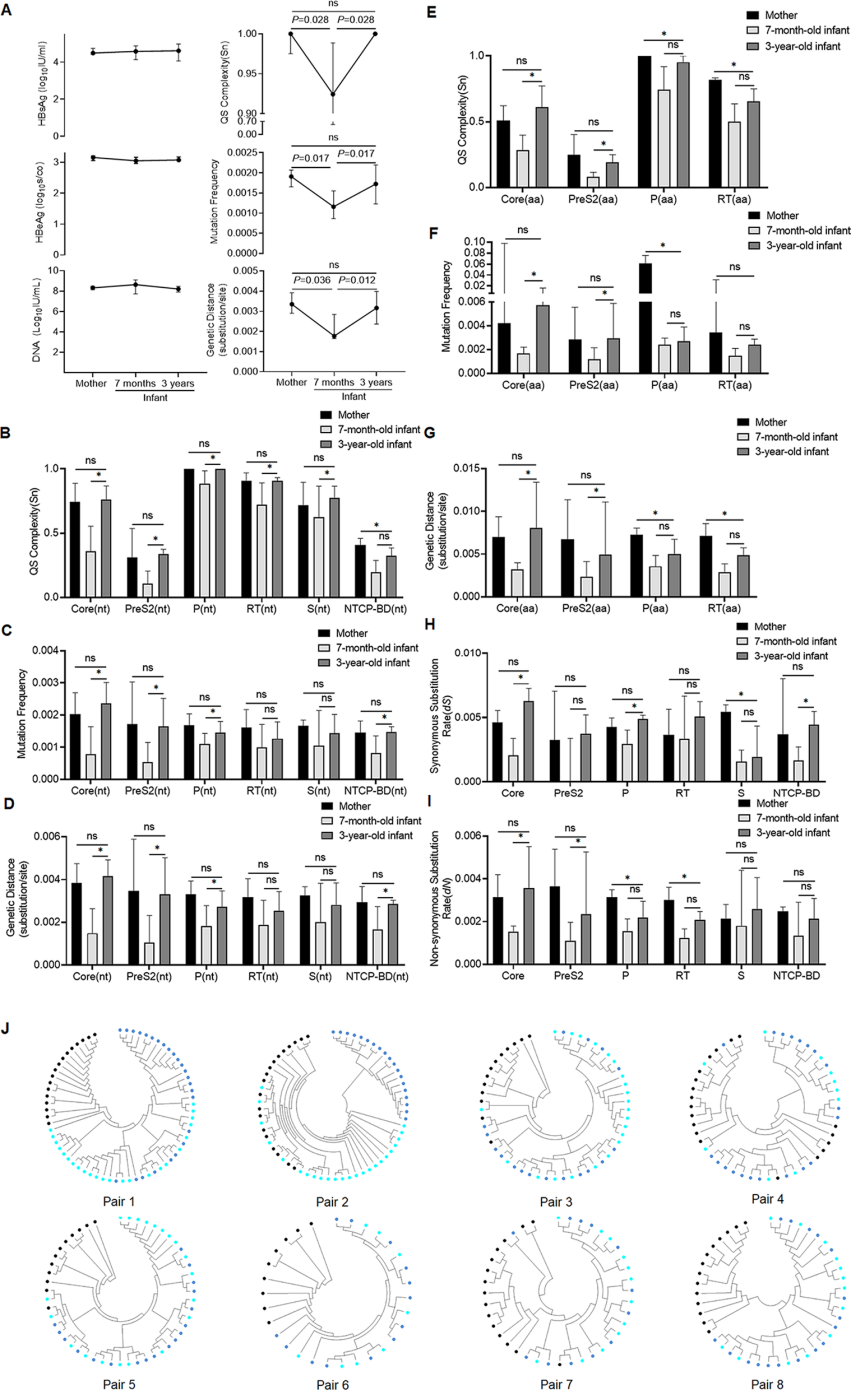
Comparative analysis of serum markers and HBV quasispecies characteristics between 8 pairs of mothers and infants at 7 months and 3 years old. Dynamic change of serum markers and HBV quasispecies characteristics at full-length HBV genome level (**A**). The quasispecies complexities (**B**), mutation frequencies (**C**) and genetic distances (**D**) of Core, PreS2, P, RT, S and NTCP-BD regions at nucleotide level at three time points. The quasispecies complexities (**E**), mutation frequencies (**F**) and genetic distances (**G**) of Core, PreS2, P and RT regions at amino acid level at three time points. The dynamic change of synonymous substitution rates (*dS*) (**H**) and the non-synonymous substitution rates (*dN*) (**I**) of Core, PreS2, P, RT, S and NTCP-BD regions at three time points. The phylogenetic trees of 8 mothers and their paired infants at 7 months and 3 years old. (**J**) Black point represents the clones from mothers, light blue point for 7-month-old infants and dark blue point for 3-year-old infants. *represents *P* < 0.05. “nt” represents nucleotide level. “aa” represents amino acid level. NTCP-BD: sodium taurocholate cotransporting polypetide binding domain; Quasispecies complexity was measured using normalized Shannon entropy (Sn). Genetic distance was calculated at the nucleotide level under Tamura 3-parameter method and at the amino acid level under Jones–Taylor–Thornton matrix-based method. dS and dN were calculated under modified Nei–Gojobori model with Jukes–Cantor correction

The quasispecies characteristic values of full-length decreased significantly after MTCT and increased to near maternal level from 7 months to 3 years old, while the levels of serum HBV DNA, HBsAg and HBeAg did not change obviously (Fig. 2A).

At nucleotide acid level, the quasispecies complexities, mutation frequencies and genetic distances of Core, PreS2 and other regions increased to near the maternal level at 3 years of age (Additional file 1: Table S4 and Fig. 2B–D). For amino acid level, the phenomenon was noticed in Core and PreS2 regions (Figs. 2E–G and Additional file 1: Table S5). The synonymous substitution rates of Core, P and NTCP-BD regions (Fig. 2H and Additional file 1: Table S6) and the non-synonymous substitution rates of Core and PreS2 regions also significantly increased to near the maternal level at 3 years old (Fig. 2I and Additional file 1: Table S6).

The phylogenetic tree of each pair revealed that most sequences of infants at 7 months and 3 years old partially mixed (Fig. 2J). The mutation rates of single nucleotide site were analyzed, and 19 mutants with mutation rates significantly changed have been found in 5 infants (Table 3). Among them, the mutation rates of 3 sites significantly decreased from 7 months to 3 years old, and caused amino acid substitutions; While in 15 nucleotide sites with the mutation rates significantly increased, 13 of them caused amino acid substitution, including 10 located at Core region. Core region is the most diverse region during HBV quasispecies evolution in the early stage of infection. It is worth noticing that these significant changing mutants in Core region were found in 4 of 5 male infants at 3 years old.

**Table 3:** The detailed nucleotide sites with mutation rate changed significantly from 7 months to 3 years of age

Case	Gender	Mutant sites	MR (7 months)	MR (3 years)	MR change	*P*	Amino acid mutation
Pair 1	Male	G375T	0.38	0.04	− 0.34	0.011	sW74L
		G1613A	0.00	0.44	0.44	< 0.001	pR841K
		G1899A	0.00	0.60	0.60	< 0.001	precG29D
		T1938C	0.00	0.28	0.28	0.01	cV13A
		T1961G	0.00	0.24	0.24	0.022	cF21C
		C2102T	0.46	0.04	− 0.42	0.001	non
		C2288A	0.00	0.68	0.68	< 0.001	cP130T
Pair 2	Male	C339A	0.58	1.00	0.42	0.004	sP62L
		T1938C	0.00	0.84	0.84	< 0.001	cV13A
		A2119C	0.00	0.37	0.37	0.002	non
		A2159G	0.00	0.58	0.58	< 0.001	cS87G
		A2189C	0.00	1.00	1.00	< 0.001	cI97L
		C2198A	0.00	0.42	0.42	0.001	cL100I
		C2288A	0.42	1.00	0.58	< 0.001	cP130T
		T2555C	0.42	1.00	0.58	< 0.001	non
		A2590T	0.42	1.00	0.58	< 0.001	pY95F
Pair 3	Male	C2381A	0.00	0.24	0.24	0.029	cP161T
Pair 4	Female	C1826T&A1827C	0.40	0.00	− 0.40	0.008	precH5S;xT152P
Pair 5	Male	C2366A	0.50	0.17	− 0.33	0.031	cP156T

MR: Mutation rate at single nucleotide site

### Dynamics of the potential NAs-resistant mutations in RT region and the mutation rates of single amino acid site in core and PreS2 regions

**Fig. 3:**
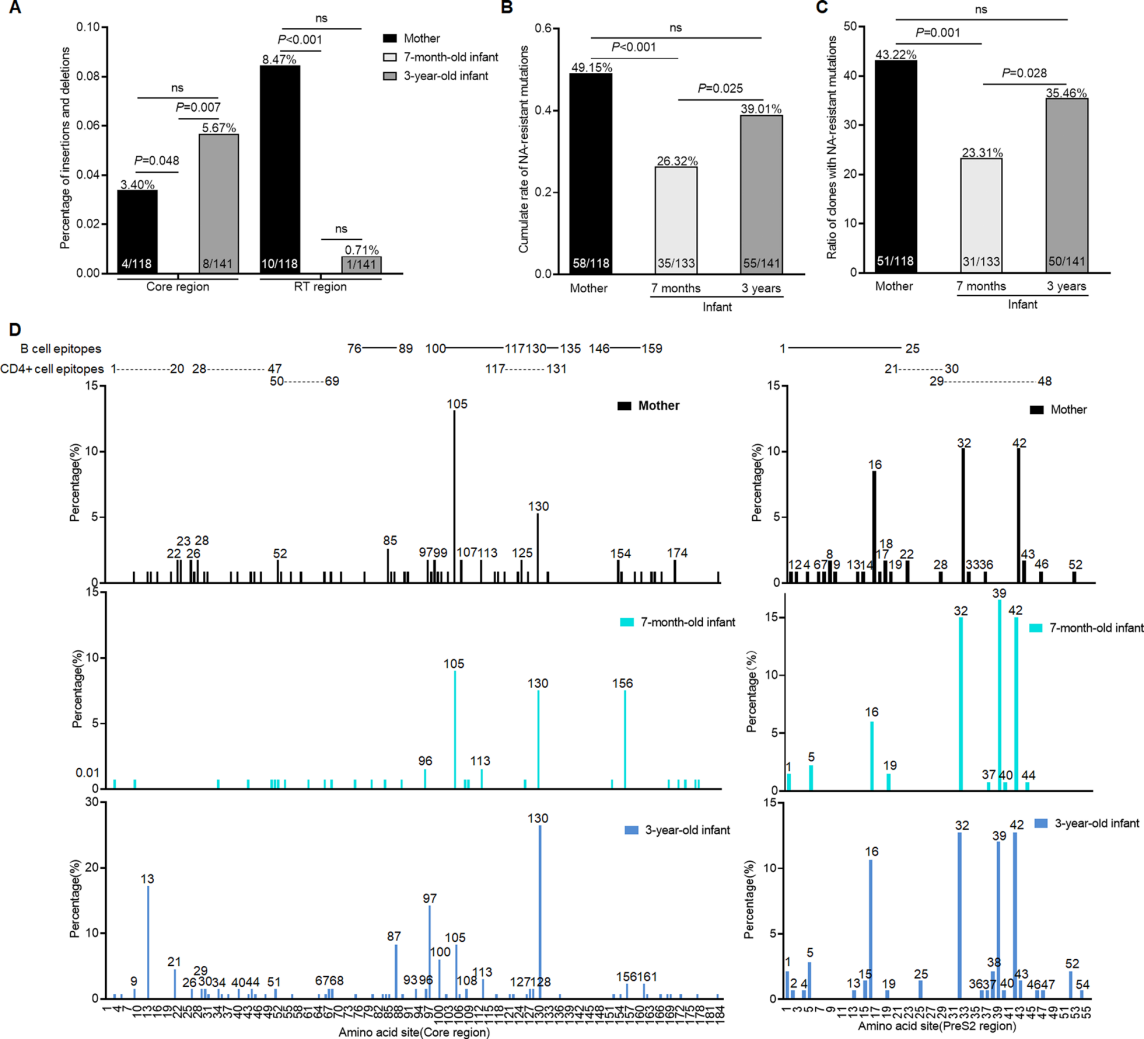
Comparative analysis of the potential NAs-resistant mutations in RT region and the mutation rates of single amino acid site in Core and PreS2 regions. Dynamic changes of the indel mutation rates in Core and RT regions of HBV genome from mothers and infants at 7 months and 3 years old. (**A**) The cumulative rate of NAs-resistant mutations in RT region of HBV genome from mothers and infants at 7 months and 3 years old. (**B**) The ratio of clones with NAs-resistant mutations in RT region of HBV genome from mothers and infants at 7 months and 3 years old. (**C**) The single amino acid site mutation rate in Core and PreS2 region of all clones from mothers and infants at 7 months and 3 years old. (**D**) Black line represents the data from mothers, light blue line for 7-month-old infants and dark blue line for 3-year-old infants. Sites with mutation rate > 1% in Core region and sites with mutation rate > 0.5% in PreS2 region were noted. All the mutations were defined based on a same consensus sequence synthesized by all clones from mothers

The potential NAs-resistant mutants defined by previous reports [24, 25] were searched from the clones of 8 mothers (118 clones) and their paired infants at 7 months (133 clones) and 3 years old (141 clones). The deletion rate in RT region was significantly higher in mothers (8.47%, 10/118) than that in 7-month-old infants (0, 0/133) (*P* < 0.001) (Fig. 3A). Totally, 21 potential NAs-resistant mutants were found and listed in Additional file 1: Table S7. The cumulative rate of NAs-resistant mutations (the number of clone with NAs-resistant mutants/total clone number, clones contain multiple NAs-resistant mutants were counted multiple times) was significantly lower in 7-month-old infants (26.32%, 35/133) than that in mothers (49.15%, 58/118) (*P* = 0.001) and 3-year-old infants (39.01%, 55/141) (*P* = 0.025), whereas there was no significant difference between mothers and 3-year-old infants (Fig. 3B). Similarly, the ratio of clones with NAs-resistant mutants (clones contain multiple NAs-resistant mutations were counted only once) was also significantly lower in 7-month-old infants (23.31%, 31/133) than that in mothers (43.22%, 51/118) (*P* = 0.001) and 3-year-old infants (35.46%, 50/141) (*P* = 0.028), whereas there was no significant difference between mothers and 3-year-old infants (Fig. 3C).

Due to the dramatic change of diversity in Core and PreS2 regions during MTCT and early infancy, the substitution rates of single amino acid site were calculated in these regions. More indels were found in mothers (3.40%, 4/118) than that in 7-month-old infants (0, 0/133) (*P* = 0.048), and there were also more indels in 3-year-old infants (5.67%, 8/141) than that in 7-month-old infants (*P* = 0.007), whereas there was no significant difference between mothers and 3-year-old infants (Fig. 3A). As shown in Fig. 3D, more substitutions in B cell and CD4+ T cell epitopes in Core and PreS2 regions were found in the clones from mothers and 3-year-old infants than that in 7-month-old infants. For 184 amino acid sites in Core region, the ratio of sites with substitution rate over 1% was lower in 7-month-old infants (2.72%, 5/184) than that in mothers (8.15%, 15/184) (*P* = 0.021) and 3-year-old infants (13.59%, 25/184) (*P* < 0.001) (Fig. 3D). For 55 amino acid sites in PreS2 region, the ratio of sites with substitution rate over 0.5% was lower in 7-month-old infants (18.18%, 10/55) than that in mothers (40%, 22/55) (*P* = 0.012) and 3-year-old infants (38.18%, 21/55) (*P* = 0.02) (Fig. 3D).

## Discussion

In our previous study, the results of full-length HBV genome clone-based sequencing showed superiority in comprehensively depicting the feature of HBV quasispecies from whole genome landscape [19]. In this study, the infants and mothers both were antiviral treatment-naive, thus these subjects were suitable for exploring HBV quasispecies’ natural dynamic changes and its interaction with host.

MTCT is considered as a bottleneck event for HBV proliferation, therefore the viral population is expected to have competent replication capacity and low mutation rate after MTCT [19]. Therefore, HBV quasispecies characteristic values were significantly decreased after MTCT, especially for the regions related with immune escape (PreS2 and Core regions) and replication capacity (RT, ENI, SPI and XP regions). Further, the decline of many substitutions in B cell and CD4+ T cell epitopes of PreS2 and Core regions after MTCT, might due to the immature immunity of infants. Since the virus strains of immune escape commonly accompanied with replication capacity undermined, the virus with these mutants might be outnumbered by wild type which have stronger replication capacity in a new environment with feeble immunity. Meanwhile, the different human leukocyte antigen (HLA) types might also play an important role during this period as the individualized mutants found in 84.62% (11/13) mother-infant pairs (Additional file 1: Table S4). There were 18 mutations with an increased mutation rate after MTCT, and 12 of them lead to amino acid substitution, including 4 substitutions in S region (P62L, W74L, W165S and S193L), 3 substitutions in PreS2 region (A11T, V17E and A39V), 5 substitutions in PreS1 (V90A), P (Y95F), PreC (H5S), Core (P156T) and X region (V5M), respectively. However, these substitutions are individualized, and none of them was found in two or more infants. Since these substitutions are mainly distributed in PreS1, PreS2 and S region (66.67%, 8/12), it indicates the select pressure on HBV surface antigen. It’s worth noticing that no substitutions were found in “a” determinant region which was considered as the major region occurring vaccine escape mutants [26]. This result was consistent with our previous report that a more complex mutant spectrum in “a” determinant region might be more vulnerable to extinct through MTCT, and the vaccine-escape mutations was not a significant factor of immunoprophylaxis failure [19].

In this study, we found that the characteristics of HBV quasispecies in Core, PreS2, P, NTCP-BD and RT regions, which were strongly correlated with the host-immunity, virus infection and replication capacity, increased to near the maternal level at 3 years old, suggested that the complexity and diversity of HBV quasispecies increased along with age and reached at adult’s level at 3 years old. Several studies have reported a negative correlation between antiviral efficacy and quasispecies complexities in certain regions, such as RT region for HBV and HVR-1 region for hepatitis C virus (HCV) [15–18, 27, 28]. In this study, both the quasispecies complexity at nucleotide level and the ratios of potential NAs-resistant mutation in RT region were lower in the 7-month-old infants than that in mothers, and increased significantly to near the maternal levels at 3 years old. Therefore, during HBV evolution in infancy, the ability of HBV quasispecies against antiviral treatment increased along with age, especially for NAs treatment, which could explain the phenomenon that more efficacious outcomes were observed in infants younger than 1 year old and NAs treatment was more effective than IFN for infants [14]. Further, more mutations in B cell and CD4+ T cell epitopes in Core and PreS2 regions were found in older infants, suggesting that it might be easier for HBV quasispecies to achieve immune clearance in infants at 7 months old than that in infants at 3 years old in term of virological factors. Combined with the fast division of liver cells in infants, the virus might be diluted, which would ultimately accelerate HBV elimination [29]. Thus, these findings could explain the phenomenon that the younger infants are more responsive to antiviral treatment [13].

## Conclusions

**Fig. 4:**
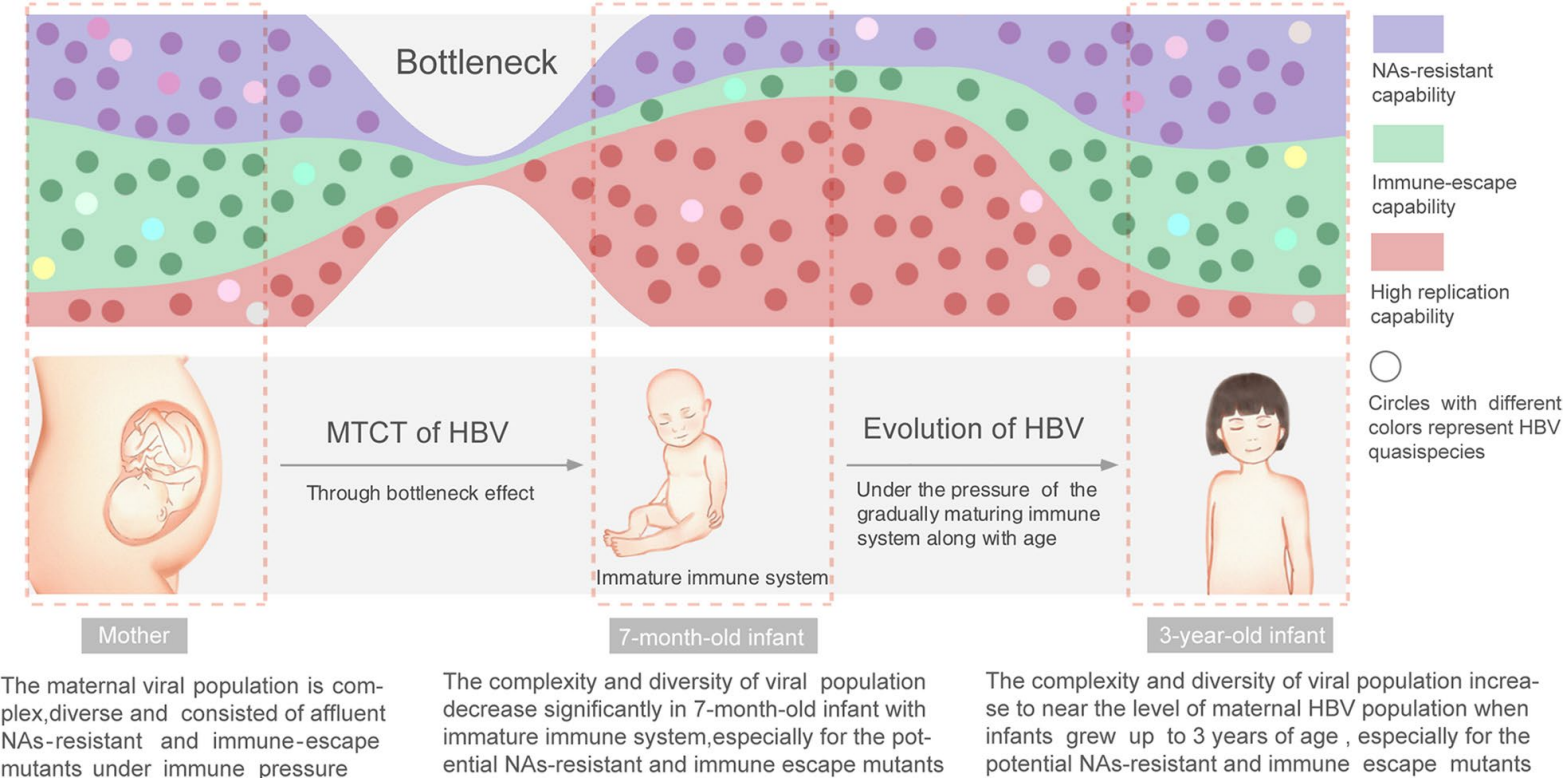
Graphic abstract for the dynamics of hepatitis B virus quasispecies after MTCT and evolution in infancy

As presented in Fig. 4, our results demonstrate a relatively simple and pure viral population with low level of potential NAs-resistant and immune-escape mutants in HBV immunoprophylaxis failure infants at 7 months old, and the viral population will grow diverse to reach the maternal level at 3 years old. This study uncovered the evolution of HBV quasispecies in infancy after mother-to-child transmission, which may provide the virological evidence for explaning that younger children are more responsive to antiviral therapy.

## Supplementary Information

**Additional file 1**. Detailed materials and supplementary Tables and Figures.

## Data Availability

Data are available upon reasonable request.

## Abbreviations

HBV: Hepatitis B virus
MTCT: Mother-to-child transmission
HCC: Hepatocellular carcinoma
CHB: Chronic hepatitis B
HBIG: Hepatitis B immunoglobin
NTCP-BD: Sodium taurocholate cotransporting polypetide binding domain
MHR: Major hydrophilic region
NAs: Nucleotide analogues
HLA: Human leukocyte antigen
