# Supplementary material for: The dynamic changes of HBV quasispecies diversity in infancy after immunoprophylaxis failure: a prospective cohort study
Source: Virol J. 2021 Nov 29;18:236. doi: 10.1186/s12985-021-01707-9 (PMC8628401; doi:10.1186/s12985-021-01707-9)
Supplement: Supplementary file 1 — Additional file 1. Detailed materials and supplementary Tables and Figures. [file 12985_2021_1707_MOESM1_ESM.docx]

**The dynamic changes of HBV quasispecies diversity in infancy after immunoprophylaxis failure: a prospective cohort study**

**Authors:** Yi Li^1^, Yiwei Xiao^1^, Lili Li^1^, Yarong Song^1^, Xiangjun Zhai^2^, Jianxun Liu^3^, Zhongping Duan^4^, Ling Yan^1^, Feng Ding^1^, Jia Liu^1^, Hui Zhuang^1^, Liguo Zhu^2^, Jie Jiang^2^, Huaibin Zou^4^, Lingxiang Li^5^, Caihong Liang^6^, Jie Wang^1^*, Jie Li^1^*

**Material and methods**

**Amplification, cloning and sequencing**

Clone samples were sequenced by 7 primers (T7 sequence and SP6 sequence in vector, H1 [5′- CGAGAAAGTGAAAGCCTGCT] [nt 1101 to 1082], H3 [5′-CTGTTGTTAGACGACGAGGCA] [nt 2340 to 2360], H5 [5′-GTGCCATTTGTTCAGTGGTTCG] [nt 683 to 704], PR3 [5′-CATAGCAGCAGGATGAAGAGGA] [nt 423 to 402], HBAS-4V [5′-ATAGGGGCATTTGGTGGTCT] [nt 2316 to 2297]).

**Sequence analyses**

Sequence segments were assembled to full-length HBV genome using the Contig-Express software (New York, USA) and Codon Code Aligner software (Centerville, MA, USA). Sequence alignment and viral quasispecies analyses were performed by MEGAX software.^1^ The following regions were aligned with the reference sequences (genotype C, GenBank accession no. AB014378 and genotype C, GenBank accession no. AB048705) using Clustal X (V.2.0) software: “a” determinant, nt 524–595; MHR region, nt 449-661; Core region, nt 1901–2452; Polymerase region (P), nt 2307–1623; NTCP-binding domain (NTCP-BD), nt 2851–3072; PreC region, nt 1814–1900; PreS1 region, nt 2848–3204; PreS2 region, nt 3205–154; RT region, nt 130–1161; S region, nt 155–835; HBx protein region (X), nt 1374–1838; Enhancer I (EN I), nt 957–1361; Enhancer II (EN II), nt 1685–1773; Basic core promoter (BCP), nt 1742–1849; Core promoter (CP), nt 1613–1849; S promoter I (SP I), 2718–2808; S promoter II (SP II), nt 2983–3210; X promoter (XP), nt 1171–1361.^2^

**Supplementary Table S1. Quasispecies characteristics of mothers and 7-month-old infants at nucleotide level**

| Regions | Mothers | 7-month-old infants | *P* |
| --- | --- | --- | --- |
| Full-length-MF | 0.002044(0.001552-0.006974) | 0.001192(0.000156-0.001586) | 0.002 |
| Full-length-Sn | 1(0.898227-1) | 0.939794(0.291727-1) | 0.016 |
| Full-length-d | 0.003786(0.002486-0.004529) | 0.001786(0.000311-0.003015) | 0.003 |
| "a" determinant-MF | 0.000965(0-0.002845) | 0.000614(0-0.001689) | 0.060 |
| "a" determinant-Sn | 0.097504(0-0.273907) | 0.059820(0-0.273908) | 0.136 |
| "a" determinant-d | 0.002012(0-0.005945) | 0.001276(0-0.003520) | 0.060 |
| MHR-MF | 0.001878(0-0.003689) | 0.000854(0-0.001677) | 0.055 |
| MHR-Sn | 0.376784(0-0.558941) | 0.177325(0-0.376784) | 0.039 |
| MHR-d | 0.003781(0-0.005880) | 0.001718(0-0.002757) | 0.033 |
| Core-MF | 0.002508(0.001066-0.020180) | 0.000906(0-0.002174) | 0.005 |
| Core-Sn | 0.796453(0.589429-0.962479) | 0.372772(0-0.772654) | 0.002 |
| Core-d | 0.004115(0.002124-0.007261) | 0.001664(0-0.003091) | 0.003 |
| NTCP-BD-MF | 0.001502(0.001060-0.030566) | 0.001126(0-0.002252) | 0.009 |
| NTCP-BD-Sn | 0.409223(0.232906-0.759176) | 0.249128(0-0.593260) | 0.039 |
| NTCP-BD-d | 0.003019(0.002136-0.008692) | 0.001861(0-0.004476) | 0.028 |
| P-MF | 0.001973(0.001231-0.004907) | 0.001075(0.000198-0.001619) | 0.003 |
| P-Sn | 1(0.898227-1) | 0.889471(0.291727-1) | 0.016 |
| P-d | 0.003833(0.002460-0.004328) | 0.001842(0.000395-0.003098) | 0.003 |
| PreC-MF | 0.001642(0.000605-0.011494) | 0(0-0.010345) | 0.345 |
| PreC-Sn | 0.155403(0-0.286086) | 0(0-0.409691) | 0.239 |
| PreC-d | 0.002740(0-0.007082) | 0(0-0.014808) | 0.583 |
| PreS1-MF | 0.001634(0.000800-0.021008) | 0.001120(0-0.0023700 | 0.075 |
| PreS1-Sn | 0.501099(0.409223-0.856864) | 0.347654(0-0.758664) | 0.116 |
| PreS1-d | 0.003260(0.001606-0.005950) | 0.002248(0-0.0046960 | 0.152 |
| PreS2-MF | 0.002020(0.000932-0.006061) | 0.000466(0-0.001818) | 0.003 |
| PreS2-Sn | 0.380897(0.207044-0.633983) | 0.108515(0-0.408431) | 0.004 |
| PreS2-d | 0.004086(0.001877-0.009482) | 0.001155(0-0.003660) | 0.003 |
| RT-MF | 0.001696(0.001314-0.002423) | 0.000929(0.000242-0.002140) | 0.019 |
| RT-Sn | 0.901158(0.776796-1) | 0.730131(0.181188-0.938614) | 0.006 |
| RT-d | 0.003406(0.002392-0.004676) | 0.001619(0.000485-0.003987) | 0.009 |
| S-MF | 0.001783(0.000979-0.029369) | 0.001028(0.000232-0.002509) | 0.033 |
| S-Sn | 0.730131(0.556339-1) | 0.612122(0.181188-0.913578) | 0.028 |
| S-d | 0.003382(0.001936-0.005019) | 0.001964(0.000466-0.004570) | 0.028 |
| X-MF | 0.002458(0.000662-0.005680) | 0.001075(0.000196-0.003656) | 0.023 |
| X-Sn | 0.717191(0.309453-0.898227) | 0.509663(0.118940-0.939794) | 0.033 |
| X-d | 0.003251(0.001328-0.007640) | 0.002134(0.000392-0.006200) | 0.046 |
| BCP-MF | 0.001984(0.000483-0.018888) | 0.000926(0-0.010185) | 0.133 |
| BCP-Sn | 0.208956(0-0.521934) | 0.131653(0-0.616040) | 0.424 |
| BCP-d | 0.002674(0-0.008661) | 0.001870(0-0.015685) | 0.657 |
| CP-MF | 0.002234(0.000649-0.009392) | 0.000879(0-0.005063) | 0.055 |
| CP-Sn | 0.376784(0.158760-0.758039) | 0.266528(0-0.713727) | 0.173 |
| CP-d | 0.003037(0.000945-0.006895) | 0.001751(0-0.007938) | 0.311 |
| EnⅠ-MF | 0.001728(0.001058-0.002989) | 0.000741(0-0.001646) | 0.004 |
| EnⅠ-Sn | 0.677862(0.210758-0.901158) | 0.383343(0-0.631325) | 0.019 |
| EnⅠ-d | 0.003477(0.002067-0.005668) | 0.001564(0-0.002894) | 0.005 |
| EN Ⅱ-MF | 0.002365(0-0.018506) | 0.000864(0-0.003745) | 0.028 |
| EN Ⅱ-Sn | 0.179130(0-0.409223) | 0.105729(0-0.408431) | 0.182 |
| EN Ⅱ-d | 0.003999(0-0.006507) | 0.001743(0-0.007723) | 0.272 |
| SPⅠ-MF | 0.002313(0-0.003925) | 0.000785(0-0.002198) | 0.012 |
| SPⅠ-Sn | 0.232906(0-0.456357) | 0.115431(0-0.259590) | 0.028 |
| SPⅠ-d | 0.003930(0-0.007704) | 0.001853(0-0.004450) | 0.019 |
| SPⅡ-MF | 0.001462(0.000516-0.002308) | 0.001096(0-0.002924) | 0.422 |
| SPⅡ-Sn | 0.376784(0.156627-0.650515) | 0.259590(0-0.677590) | 0.133 |
| SPⅡ-d | 0.002947(0.001036-0.004601) | 0.002207(0-0.005326) | 0.311 |
| XP-MF | 0.001571(0.000436-0.002992) | 0.000785(0-0.001571) | 0.009 |
| XP-Sn | 0.307701(0-0.631325) | 0.196113(0-0.408431) | 0.023 |
| XP-d | 0.003159(0-0.006034) | 0.001580(0-0.003159) | 0.011 |

MF: Mutation Frequency; Sn: Shannon entropy; d: Genetic distance

**Supplementary Table S2. Quasispecies characteristics of mothers and 7-month-old infants at amino acid level**

| Regions | Mothers | 7-month-old infants | *P* |
| --- | --- | --- | --- |
| "a" determinant-MF | 0(0-0.006579) | 0.001736(0-0.004167) | 0.646 |
| "a" determinant-Sn | 0(0-0.207044) | 0.059820(0-0.141182) | 0.575 |
| "a" determinant-d | 0(0-0.013897) | 0.003659(0.000001-0.008995) | 0.861 |
| MHR-MF | 0.003707(0-0.031915) | 0.001921(0-0.047284) | 0.182 |
| MHR-Sn | 0.232906(0-0.456357) | 0.177325(0-0.376784) | 0.239 |
| MHR-d | 0.006390(0.000001-0.010977) | 0.004049(0.000001-0.008732) | 0.480 |
| Core-MF | 0.092391(0.001279-0.195652) | 0.001630(0-0.012934) | 0.006 |
| Core-Sn | 0.589429(0.156627-0.929897) | 0.279320(0-0.502311) | 0.009 |
| Core-d | 0.007893(0.001958-0.013026) | 0.003149(0.000001-0.006141) | 0.004 |
| NTCP-BD-MF | 0.003180(0.000795-0.342664) | 0.001689(0-0.026988) | 0.015 |
| NTCP-BD-Sn | 0.273908(0.078963-0.409223) | 0.131653(0-0.464770) | 0.133 |
| NTCP-BD-d | 0.006267(0.001743-0.011960) | 0.003027(0.000001-0.009100) | 0.152 |
| P-MF | 0.067466(0.002300-0.336578) | 0.002610(0.000148-0.049947) | 0.006 |
| P-Sn | 1(0.898227-1) | 0.753547(0.177325-1) | 0.004 |
| P-d | 0.007471(0.005090-0.008573) | 0.003193(0.000302-0.006058) | 0.002 |
| PreC-MF | 0.006897(0-0.103448) | 0(0-0.044061) | 0.091 |
| PreC-Sn | 0.090445(0-0.227810) | 0(0-0.409691) | 0.530 |
| PreC-d | 0.004952(0-0.017386) | 0.000001(0.000001-0.030142) | 0.507 |
| PreS1-MF | 0.002801(0.001867-0.331933) | 0.002101(0-0.028037) | 0.046 |
| PreS1-Sn | 0.336829(0.192924-0.409223) | 0.317891(0-0.549744) | 0.463 |
| PreS1-d | 0.005079(0.002575-0.007434) | 0.004396(0.000001-0.009482) | 0.196 |
| PreS2-MF | 0.003896(0.001299-0.059394) | 0.000758(0-0.004546) | 0.006 |
| PreS2-Sn | 0.286086(0.105729-0.582300) | 0.097504(0-0.277528) | 0.004 |
| PreS2-d | 0.007832(0.002690-0.019904) | 0.002496(0.000001-0.007281) | 0.006 |
| RT-MF | 0.004199(0.002394-0.119298) | 0.001575(0.000363-0.036907) | 0.028 |
| RT-Sn | 0.807835(0.639472-0.962479) | 0.456688(0.139062-0.849915) | 0.001 |
| RT-d | 0.007777(0.003832-0.010304) | 0.002725(0.000745-0.007271) | 0.002 |
| S-MF | 0.004091(0.002056-0.111958) | 0.002019(0.000551-0.025502) | 0.116 |
| S-Sn | 0.593803(0.350656-0.929897) | 0.533109(0.181188-0.901158) | 0.101 |
| S-d | 0.006018(0.003835-0.010673) | 0.004038(0.000512-0.012666) | 0.221 |
| X-MF | 0.006989(0.000993-0.031879) | 0.002037(0.000587-0.007742) | 0.016 |
| X-Sn | 0.466990(0-0.796453) | 0.328334(0.118940-0.856864) | 0.196 |
| X-d | 0.007176(0-0.011488) | 0.003873(0.001211-0.014359) | 0.279 |

MF: Mutation Frequency; Sn: Shannon entropy; d: Genetic distance

**Supplementary Table S3. Synonymous substitution and non-synonymous substitution rate of mothers and 7-month-old infants**

| Regions | Mothers | 7-month-old infants | *P* |
| --- | --- | --- | --- |
| a determinant*-dS* | 0.006513(0-0.012378) | 0(0-0.006189) | 0.018 |
| a determinant*-dN* | 0(0-0.005806) | 0.001534(0-0.003648) | 0.959 |
| MHR*-dS* | 0.005448(0-0.012806) | 0(0-0.005782) | 0.005 |
| MHR*-dN* | 0.002696(0-0.004640) | 0.001715(0-0.003658) | 0.530 |
| Core*-dS* | 0.005090(0.003006-0.011065) | 0.002354(0-0.006079) | 0.005 |
| Core*-dN* | 0.003545(0.000898-0.005841) | 0.001475(0-0.002763) | 0.004 |
| NTCP-BD*-dS* | 0.005661(0-0.022129) | 0.001639(0-0.008502) | 0.012 |
| NTCP-BD*-dN* | 0.002489(0.000693-0.004725) | 0.001207(0-0.003633) | 0.152 |
| P*-dS* | 0.004349(0.002657-0.006436) | 0.002887(0.001178-0.004929) | 0.009 |
| P*-dN* | 0.003252(0.002206-0.003737) | 0.001401(0.000132-0.002652) | 0.002 |
| PreC*-dS* | 0(0-0.013303) | 0(0-0.007559) | 0.310 |
| PreC*-dN* | 0.002111(0-0.009733) | 0(0-0.019578) | 0.583 |
| PreS1*-dS* | 0.005815(0-0.015274) | 0.003558(0-0.007838) | 0.133 |
| PreS1*-dN* | 0.002149(0.001091-0.003145) | 0.001858(0-0.004014) | 0.196 |
| PreS2*-dS* | 0.003510(0-0.025175) | 0(0-0.004806) | 0.019 |
| PreS2*-dN* | 0.004622(0.001284-0.009456) | 0.001184(0-0.003401) | 0.006 |
| RT*-dS* | 0.003477(0.001271-0.008357) | 0.003422(0.000795-0.007697) | 0.600 |
| RT*-dN* | 0.003272(0.001612-0.004340) | 0.001176(0.000321-0.003129) | 0.002 |
| S*-dS* | 0.005098(0.002416-0.008361) | 0.001497(0-0.004209) | 0.002 |
| S*-dN* | 0.002552(0.001612-0.004508) | 0.001638(0.000208-0.005292) | 0.196 |
| X*-dS* | 0.004172(0.000874-0.018359) | 0.003034(0-0.007200) | 0.016 |
| X*-dN* | 0.003195(0-0.005124) | 0.001701(0.000532-0.006270) | 0.249 |

*dS*: synonymous substitution rate; *dN*: non-synonymous substitution rate

**Supplementary Table S4. Quasispecies characteristics of 8 pairs of mothers, 7-month-old and 3-year-old infants at nucleotide level**

| Regions | Mothers | 7-month-old infants | 3-year-old infants | *P1* | *P2* |
| --- | --- | --- | --- | --- | --- |
| Full-length-MF | 0.001907  (0.001552-0.002557) | 0.001160  (0.000156-0.001586) | 0.001724  (0.0003111-0.002423) | 0.484 | 0.017 |
| Full-length-Sn | 1  (0.965872-1) | 0.924459  (0.353759-1) | 1  (0.466168-1) | 0.713 | 0.028 |
| Full-length-d | 0.003351  (0.002486-0.004079) | 0.001774  (0.000311-0.003015) | 0.003167  (0.000575-0.004273) | 0.889 | 0.012 |
| "a" determinant-MF | 0.001014  (0-0.002845) | 0.000827  (0-0.001689) | 0.000987  (0-0.003655) | 0.866 | 0.753 |
| "a" determinant-Sn | 0.102938  (0-0.273908) | 0.082775  (0-0.161765) | 0.093739  (0-0.280666) | 0.499 | 0.500 |
| "a" determinant-d | 0.002110  (0-0.005945) | 0.001721  (0-0.003520) | 0.002003  (0-0.006936) | 0.612 | 0.500 |
| MHR-MF | 0.001994  (0.000626-0.002347) | 0.000896  (0.000196-0.001565) | 0.000991  (0-0.002224) | 0.128 | 0.753 |
| MHR-Sn | 0.371077  (0.179130-0.466990) | 0.188651  (0.054500-0.336829) | 0.249828  (0-0.469603) | 0.401 | 0.161 |
| MHR-d | 0.004024  (0.001257-0.004769) | 0.001804  (0.000393-0.002757) | 0.001935  (0-0.004316) | 0.123 | 0.575 |
| Core-MF | 0.002018  (0.001066-0.003623) | 0.000782  (0-0.002174) | 0.002355  (0.000836-0.004577) | 0.484 | 0.017 |
| Core-Sn | 0.743325  (0.589429-0.929897) | 0.360213  (0-0.603309) | 0.760281  (0.386376-0.953510) | 1.000 | 0.012 |
| Core-d | 0.003839  (0.002124-0.005050) | 0.001493  (0-0.002937) | 0.004151  (0.001399-0.006353) | 0.674 | 0.012 |
| NTCP-BD-MF | 0.001462  (0.001185-0.002703) | 0.000826  (0-0.001733) | 0.001471  (0-0.002120) | 0.263 | 0.028 |
| NTCP-BD-Sn | 0.405459  (0.273908-0.556339) | 0.194788  (0-0.501099) | 0.322188  (0-0.452361) | 0.012 | 0.063 |
| NTCP-BD-d | 0.002940  (0.002387-0.005361) | 0.001660  (0-0.003484) | 0.002853  (0-0.004277) | 0.208 | 0.028 |
| P-MF | 0.001692  (0.001231-0.002150) | 0.001109  (0.000198-0.001610) | 0.001449  (0.000213-0.002044) | 0.123 | 0.025 |
| P-Sn | 1  (0.965872-1) | 0.882896  (0.353759-1) | 1  (0.406945-1) | 0.713 | 0.028 |
| P-d | 0.003317  (0.002460-0.004054) | 0.001821  (0.000395-0.002996) | 0.002735  (0.000426-0.003976) | 0.069 | 0.036 |
| PreC-MF | 0.002486  (0.000605-0.011494) | 0  (0-0.010345) | 0.001304  (0-0.005517) | 0.401 | 0.893 |
| PreC-Sn | 0.157694  (0.070028-0.286086) | 0  (0-0.409691) | 0.098057  (0-0.273908) | 0.401 | 0.465 |
| PreC-d | 0.002679  (0.001224-0.004989) | 0  (0-0.014808) | 0.001744  (0-0.007697) | 0.779 | 0.465 |
| PreS1-MF | 0.001565  (0.000989-0.001917) | 0.001027  (0-0.002370) | 0.001443  (0-0.003295) | 0.612 | 0.237 |
| PreS1-Sn | 0.484045  (0.452361-0.588415) | 0.342242  (0-0.758664) | 0.400841  (0-0.816442) | 0.208 | 0.612 |
| PreS1-d | 0.003038  (0.001983-0.003822) | 0.002063  (0-0.004696) | 0.002854  (0-0.006225) | 0.401 | 0.176 |
| PreS2-MF | 0.001723  (0.000932-0.003232) | 0.000536  (0-0.001818) | 0.001646  (0-0.003333) | 0.484 | 0.018 |
| PreS2-Sn | 0.309442  (0.207044-0.593803) | 0.107122  (0-0.408431) | 0.338204  (0-0.502311) | 0.401 | 0.018 |
| PreS2-d | 0.003479  (0.001877-0.006127) | 0.001047  (0-0.003660) | 0.003316  (0-0.006546) | 0.401 | 0.018 |
| RT-MF | 0.001610  (0.001416-0.002261) | 0.000993  (0.000242-0.002140) | 0.001273  (0.000224-0.001861) | 0.093 | 0.401 |
| RT-Sn | 0.908732  (0.829361-1) | 0.719667  (0.181188-0.938614) | 0.904165  (0.309453-0.982773) | 0.484 | 0.012 |
| RT-d | 0.003185  (0.002546-0.004541) | 0.001870  (0.000485-0.003987) | 0.002532  (0.000448-0.003553) | 0.093 | 0.327 |
| S-MF | 0.001669  (0.000979-0.002447) | 0.001048  (0.000367-0.002509) | 0.001432  (0.000226-0.002350) | 0.779 | 0.483 |
| S-Sn | 0.716386  (0.556339-1) | 0.625797  (0.181188-0.913578) | 0.774725  (0.208956-0.941817) | 0.401 | 0.049 |
| S-d | 0.003246  (0.001936-0.004918) | 0.002013  (0.000736-0.004570) | 0.002820  (0.000453-0.004404) | 1.000 | 0.263 |
| X-MF | 0.002663  (0.000662-0.005680) | 0.000978  (0.000538-0.003656) | 0.001915  (0.000331-0.002688) | 0.401 | 0.208 |
| X-Sn | 0.670344  (0.309453-0.782195) | 0.529791  (0.181188-0.939794) | 0.669752  (0.167381-1) | 0.575 | 0.093 |
| X-d | 0.002855  (0.001328-0.004638) | 0.001950  (0.001080-0.006200) | 0.003773  (0.000332-0.005338) | 0.263 | 0.123 |
| BCP-MF | 0.005107  (0.000483-0.018889) | 0.001042  (0-0.010185) | 0.001846  (0-0.003704) | 0.123 | 0.917 |
| BCP-Sn | 0.200940  (0-0.336829) | 0.146709  (0-0.616040) | 0.214510  (0-0.442114) | 0.674 | 1.000 |
| BCP-d | 0.002776  (0-0.008661) | 0.002086  (0-0.015685) | 0.003629  (0-0.007150) | 0.735 | 0.753 |
| CP-MF | 0.003190  (0.000649-0.009392) | 0.001015  (0-0.005063) | 0.002656  (0.000325-0.003165) | 0.263 | 0.161 |
| CP-Sn | 0.296893  (0.158760-0.479052) | 0.301679  (0-0.713727) | 0.446042  (0.105729-0.814038) | 0.017 | 0.123 |
| CP-d | 0.002330  (0.000945-0.005564) | 0.002032  (0-0.007938) | 0.004570  (0-0.006371) | 0.050 | 0.091 |
| EnⅠ-MF | 0.001783  (0.001235-0.002989) | 0.000864  (0-0.001646) | 0.000920  (0.000190-0.001920) | 0.036 | 0.263 |
| EnⅠ-Sn | 0.693444  (0.442114-0.901158) | 0.393003  (0-0.562831) | 0.437707  (0.105729-0.756519) | 0.093 | 0.161 |
| EnⅠ-d | 0.003588  (0.002067-0.005668) | 0.001733  (0-0.002894) | 0.001846  (0.000381-0.003726) | 0.069 | 0.208 |
| EN Ⅱ-MF | 0.001800  (0-0.018506) | 0.001134  (0-0.003745) | 0.001597  (0-0.006742) | 0.674 | 0.128 |
| EN Ⅱ-Sn | 0.138317  (0-0.273908) | 0.133747  (0-0.408431) | 0.174607  (0-0.543196) | 0.176 | 0.310 |
| EN Ⅱ-d | 0.002811  (0-0.006081) | 0.002271  (0-0.007723) | 0.003153  (0-0.013333) | 0.237 | 0.176 |
| SPⅠ-MF | 0.002334  (0.000646-0.003663) | 0.001007  (0-0.001832) | 0.001669  (0-0.003879) | 0.263 | 0.176 |
| SPⅠ-Sn | 0.241432  (0.078963-0.456357) | 0.136418  (0-0.234935) | 0.179984  (0-0.452361) | 0.401 | 0.310 |
| SPⅠ-d | 0.004054  (0.001308-0.007434) | 0.002222  (0-0.003728) | 0.003379  (0-0.007694) | 0.484 | 0.237 |
| SPⅡ-MF | 0.001424  (0.000516-0.002308) | 0.001170  (0-0.002924) | 0.001472  (0-0.003870) | 0.484 | 0.237 |
| SPⅡ-Sn | 0.338204  (0.156627-0.556340) | 0.298210  (0-0.677590) | 0.314714  (0-0.683641) | 0.889 | 0.310 |
| SPⅡ-d | 0.002868  (0.001036-0.004601) | 0.002355  (0-0.005326) | 0.002644  (0-0.007166) | 0.484 | 0.237 |
| XP-MF | 0.001305  (0.000436-0.002416) | 0.000750  (0-0.001571) | 0.001140  (0.000403-0.001745) | 0.398 | 0.123 |
| XP-Sn | 0.296893  (0-0.591494) | 0.186719  (0-0.408431) | 0.239704  (0.105729-0.380897) | 0.327 | 0.208 |
| XP-d | 0.002627  (0-0.004867) | 0.001508  (0-0.003159) | 0.002293  (0.000810-0.003373) | 0.401 | 0.123 |

MF: Mutation Frequency; Sn: Shannon entropy; d: Genetic distance

*P1* represents the comparison between mothers and 3-year-old infants; *P2* represents the comparison between 7-month-old infants and 3-year-old infants

**Supplementary Table S5. Quasispecies characteristics of 8 pairs of mothers, 7-month-old and 3-year-old infants at amino acid level**

| Regions | Mothers | 7-month-old infants | 3-year-old infants | *P1* | *P2* |
| --- | --- | --- | --- | --- | --- |
| "a" determinant-MF | 0.001389  (0-0.006579) | 0.002550  (0-0.004167) | 0.002322  (0-0.004167) | 0.917 | 0.273 |
| "a" determinant-Sn | 0  (0-0.207044) | 0.082775  (0-0.141182) | 0.074496  (0-0.115431) | 0.917 | 0.138 |
| "a" determinant-d | 0  (0-0.013897) | 0.005411  (0.000001-0.008995) | 0.004911  (0.000001-0.008522) | 0.779 | 0.225 |
| MHR-MF | 0.004201  (0.000966-0.031915) | 0.002364  (0.000587-0.003521) | 0.002237  (0-0.003521) | 0.161 | 0.917 |
| MHR-Sn | 0.250859  (0.090445-0.456357) | 0.179257  (0.054500-0.336829) | 0.181836  (0-0.336829) | 0.327 | 0.866 |
| MHR-d | 0.007027  (0.000001-0.010977) | 0.004737  (0.001239-0.007415) | 0.004121  (0.000001-0.007434) | 0.327 | 0.263 |
| Core-MF | 0.004210  (0.001279-0.114130) | 0.001680  (0-0.004348) | 0.005737  (0.002245-0.025978) | 0.575 | 0.025 |
| Core-Sn | 0.508458  (0.156627-0.929897) | 0.285524  (0-0.500000) | 0.609467  (0.386376-0.814038) | 0.327 | 0.012 |
| Core-d | 0.006994  (0.001958-0.012033) | 0.003209  (0.000001-0.006141) | 0.008039  (0.003462-0.393805) | 0.208 | 0.012 |
| NTCP-BD-MF | 0.002982  (0.001502-0.003861) | 0.001520  (0-0.004158) | 0.002477  (0-0.012811) | 0.484 | 0.176 |
| NTCP-BD-Sn | 0.269914  (0.158760-0.336829) | 0.125104  (0-0.406945) | 0.224023  (0-0.452361) | 0.484 | 0.237 |
| NTCP-BD-d | 0.006262  (0.003281-0.007412) | 0.003346  (0.000001-0.009100) | 0.005352  (0.000001-0.010458) | 0.327 | 0.237 |
| P-MF | 0.061377  (0.002300-0.104397) | 0.002391  (0.000148-0.049947) | 0.002704  (0.000182-0.026656) | 0.017 | 0.263 |
| P-Sn | 1  (0.950440-1) | 0.741839  (0.181188-1) | 0.953317  (0.209856-1) | 0.046 | 0.063 |
| P-d | 0.007228  (0.005090-0.008461) | 0.003567  (0.000302-0.005125) | 0.004987  (0.000372-0.007366) | 0.012 | 0.123 |
| PreC-MF | 0.037931  (0-0.095335) | 0  (0-0.017241) | 0.002028  (0-0.013793) | 0.091 | 0.715 |
| PreC-Sn | 0.098087  (0-0.227810) | 0  (0-0.409691) | 0.098057  (0-0.273908) | 1.000 | 0.715 |
| PreC-d | 0.005291  (0-0.012639) | 0.000001  (0.000001-0.030142) | 0.004511  (0.000001-0.019850) | 0.779 | 0.715 |
| PreS1-MF | 0.002727  (0.001867-0.016160) | 0.002101  (0-0.004525) | 0.002745  (0-0.013471) | 0.889 | 0.091 |
| PreS1-Sn | 0.324006  (0.232906-0.406945) | 0.319957  (0-0.501099) | 0.332016  (0-0.589429) | 0.779 | 0.398 |
| PreS1-d | 0.004886  (0.003115-0.007434) | 0.004400  (0.000001-0.009482) | 0.005281  (0.000001-0.010367) | 0.889 | 0.128 |
| PreS2-MF | 0.002871  (0.001299-0.059394) | 0.001205  (0-0.003636) | 0.002961  (0-0.008182) | 0.889 | 0.018 |
| PreS2-Sn | 0.246565  (0.105729-0.442114) | 0.081466  (0-0.277528) | 0.192219  (0-0.420331) | 0.484 | 0.018 |
| PreS2-d | 0.006716  (0.002690-0.013880) | 0.002335  (0.000001-0.006674) | 0.004952  (0.000001-0.015319) | 0.575 | 0.018 |
| RT-MF | 0.003441  (0.002394-0.119298) | 0.001497  (0.000363-0.003634) | 0.002408  (0-0.007913) | 0.063 | 0.263 |
| RT-Sn | 0.818598  (0.639472-0.962479) | 0.499942  (0.181188-0.698114) | 0.654804  (0-0.783055) | 0.036 | 0.263 |
| RT-d | 0.007113  (0.005856-0.009815) | 0.002856  (0.000745-0.005978) | 0.004838  (0.000001-0.007124) | 0.012 | 0.161 |
| S-MF | 0.004248  (0.002056-0.111958) | 0.002111  (0.000551-0.006695) | 0.002932  (0.000678-0.006246) | 0.263 | 0.263 |
| S-Sn | 0.539130  (0.350656-0.929897) | 0.538153  (0.181188-0.901158) | 0.613943  (0.208956-0.890135) | 0.484 | 0.208 |
| S-d | 0.005056  (0.003835-0.010673) | 0.004450  (0.001213-0.012666) | 0.006361  (0.001493-0.012630) | 0.484 | 0.327 |
| X-MF | 0.010478  (0.000993-0.031879) | 0.001747  (0.000811-0.007742) | 0.003660  (0.002329-0.005914) | 0.036 | 0.123 |
| X-Sn | 0.437632  (0-0.677862) | 0.315063  (0.161765-0.856864) | 0.544275  (0.167381-0.953510) | 0.161 | 0.123 |
| X-d | 0.005919  (0-0.008865) | 0.003510  (0.001680-0.014359) | 0.007092  (0.001037-0.012280) | 0.401 | 0.327 |

MF: Mutation Frequency; Sn: Shannon entropy; d: Genetic distance

*P1* represents the comparison between mothers and 3-year-old infants; *P2* represents the comparison between 7-month-old infants and 3-year-old infants

**Supplementary Table S6. Synonymous substitution and non-synonymous substitution rate of mothers and 7-month-old infants**

| Regions | Mothers | 7-month-old infants | 3-year-old infants | *P1* | *P2* |
| --- | --- | --- | --- | --- | --- |
| “a” determinant*-dS* | 0.003257  (0-0.010315) | 0  (0-0.005162) | 0  (0-0.023985) | 0.715 | 0.655 |
| “a” determinant*-dN* | 0  (0-0.005806) | 0.002227  (0-0.003648) | 0.002036  (0-0.003456) | 0.917 | 0.225 |
| MHR*-dS* | 0.005096  (0.002005-0.008231) | 0  (0-0.004762) | 0.002708  (0-0.009362) | 0.123 | 0.249 |
| MHR*-dN* | 0.002973  (0-0.004640) | 0.002115  (0.000524-0.003151) | 0.001755  (0-0.003147) | 0.327 | 0.263 |
| Core*-dS* | 0.004636  (0.003006-0.009909) | 0.002065  (0-0.006079) | 0.006263  (0.000836-0.008384) | 0.779 | 0.049 |
| Core*-dN* | 0.003140  (0.000878-0.005391) | 0.001522  (0-0.002745) | 0.003558  (0.001557-0.006058) | 0.401 | 0.012 |
| NTCP-BD*-dS* | 0.003712  (0.002070-0.015411) | 0.001639  (0-0.003279) | 0.004430  (0-0.012560) | 0.401 | 0.028 |
| NTCP-BD*-dN* | 0.002485  (0.001306-0.002945) | 0.001327  (0-0.003633) | 0.002123  (0-0.004165) | 0.327 | 0.237 |
| P*-dS* | 0.004254  (0.002657-0.005695) | 0.002964  (0.001178-0.004465) | 0.004886  (0.001208-0.005520) | 0.575 | 0.012 |
| P*-dN* | 0.003131  (0.002206-0.003655) | 0.001544  (0.000132-0.002250) | 0.002193  (0.000163-0.003226) | 0.012 | 0.123 |
| PreC*-dS* | 0  (0-0.0125060 | 0  (0-0.007559) | 0  (0-0.008600) | 0.715 | 0.655 |
| PreC*-dN* | 0.002273  (0-0.005319) | 0  (0-0.019578) | 0.001729  (0-0.007575) | 0.889 | 0.465 |
| PreS1*-dS* | 0.005475  (0.002720-0.009045) | 0.002012  (0-0.007838) | 0.004465  (0-0.011978) | 0.575 | 0.176 |
| PreS1*-dN* | 0.002066  (0.001328-0.003145) | 0.001858  (0-0.004014) | 0.002234  (0-0.004381) | 0.889 | 0.128 |
| PreS2*-dS* | 0.003273  (0.002400-0.009270) | 0  (0-0.004806) | 0.003743  (0-0.007238) | 0.401 | 0.116 |
| PreS2*-dN* | 0.003637  (0.001284-0.006598) | 0.001105  (0-0.003253) | 0.002345  (0-0.007242) | 0.484 | 0.018 |
| RT*-dS* | 0.003654  (0.001271-0.006454) | 0.003326  (0.000795-0.007697) | 0.005074  (0.001832-0.007326) | 0.674 | 0.208 |
| RT*-dN* | 0.003002  (0.002469-0.004143) | 0.001230  (0.000321-0.002577) | 0.002087  (0-0.003081) | 0.012 | 0.161 |
| S*-dS* | 0.005434  (0.003072-0.006125) | 0.001563  (0-0.002986) | 0.001920  (0-0.004984) | 0.036 | 0.327 |
| S*-dN* | 0.002127  (0.001612-0.004508) | 0.001801  (0.000491-0.005292) | 0.002582  (0.000604-0.005124) | 0.484 | 0.327 |
| X*-dS* | 0.004653  (0.000874-0.007155) | 0.003046  (0-0.007200) | 0.005518  (0-0.007117) | 1.000 | 0.063 |
| X*-dN* | 0.002654  (0-0.003923) | 0.001525  (0.000737-0.006270) | 0.003120  (0.000453-0.005378) | 0.401 | 0.263 |

*dS*: synonymous substitution rate; *dN*: non-synonymous substitution rate

*P1* represents the comparison between mothers and 3-year-old infants; *P2* represents the comparison between 7-month-old infants and 3-year-old infants

**Supplementary Table S7. Number of clones with potential NAr mutants**

| NAr mutations | Number of clones with deletion or mutation at this site | | |
| --- | --- | --- | --- |
|  | Mother | 7-month-old infants | 3-year-old infants |
| I169T | 2 | 0 | 1 |
| A194T | 0 | 1 | 1 |
| N236T | 0 | 0 | 2 |
| L80I/V | 2 | 0 | 0 |
| S53N | 0 | 1 | 1 |
| T54N | 1 | 0 | 0 |
| V84M | 0 | 2 | 1 |
| S85A | 0 | 2 | 1 |
| I91L | 0 | 1 | 0 |
| Y126C/R/H | 1 | 0 | 0 |
| T128I/N | 3 | 0 | 0 |
| F166L | 1 | 0 | 0 |
| V191I | 0 | 0 | 3 |
| V207I | 0 | 0 | 3 |
| V214A | 1 | 2 | 1 |
| Q215E/H/P/S | 1 | 1 | 0 |
| L217R | 0 | 0 | 1 |
| E218D | 0 | 1 | 0 |
| F221Y | 1 | 1 | 1 |
| N238D/S/T | 2 | 0 | 0 |
| S/C256G | 33 | 23 | 38 |
| deletion | 10 | 0 | 1 |
| Total number | 58 | 35 | 55 |

**Supplementary Figure S1. The phylogenetic analysis of HBV genome sequences in 13 mother-infant pairs.** (A) The neighbor-joining phylogenetic trees were constructed for the cloned full-length HBV genome sequences between 13 mothers and their paired 7-month-old infants, (B) 8 infants at 7 months and 3 years old, (C) as well as 8 3-year-old infants with their paired mothers. The red sequences represented the reference sequences of genotype B (GenBank accession No. LC057377), C (GenBank accession No. AB014378) or D HBV (GenBank accession No. LT718449). The reliability of pairwise comparison and phylogenetic tree analyses were assessed **
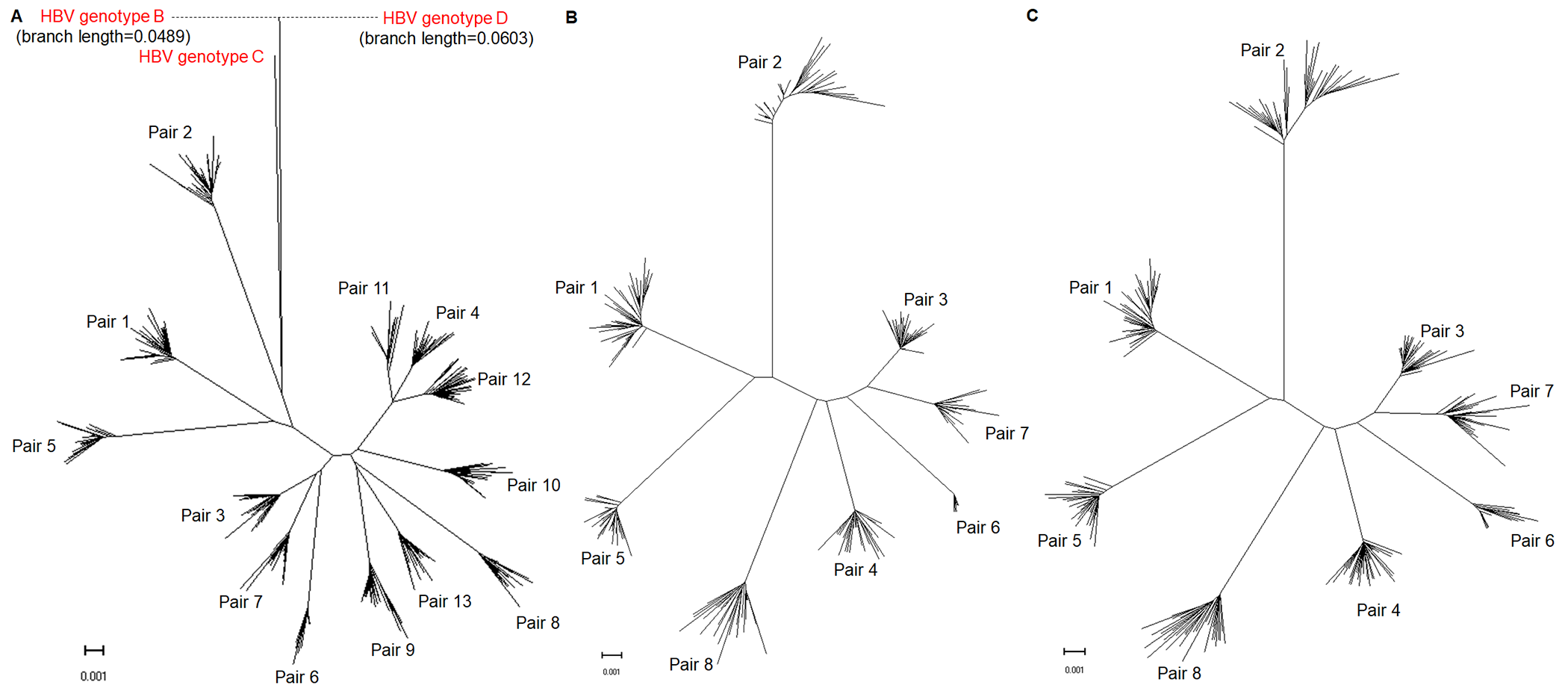
**by 1000 replicates bootstrapping.

**Supplementary Figure S2. The phylogenetic analysis of each 13 mother-infant pair.** Black point represents the full-length HBV genome clones from mothers, and light blue point represents the full-length HBV genome clones from 7-month-old infants.

**
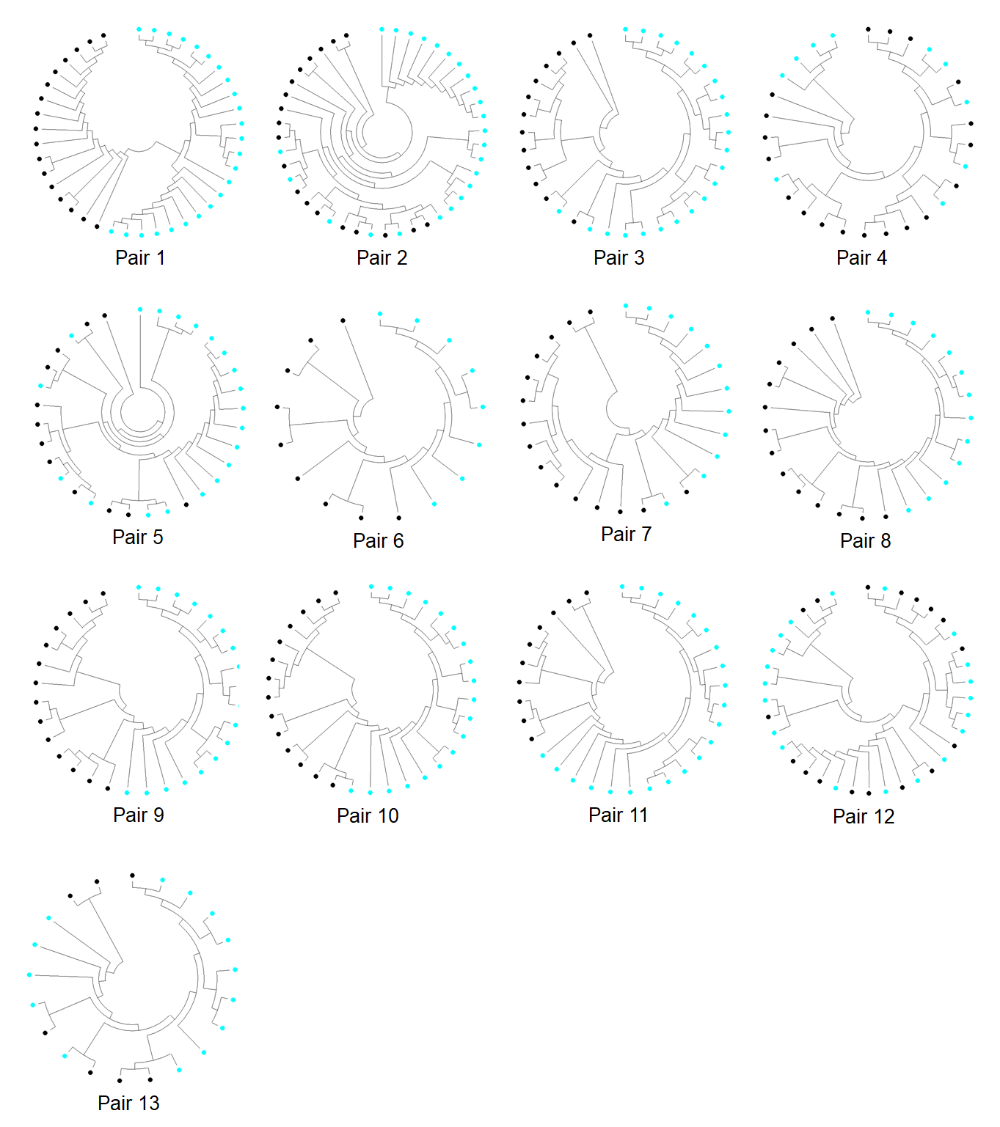
**

**References:**

1. Kumar S, Stecher G, Li M, et al. MEGA X: Molecular Evolutionary Genetics Analysis across Computing Platforms. *Mol Biol Evol* 2018;35(6):1547-49. doi: 10.1093/molbev/msy096 [published Online First: 2018/05/04]

2. Hao R, Xiang K, Peng Y, et al. Naturally occurring deletion/insertion mutations within HBV whole genome sequences in HBeAg-positive chronic hepatitis B patients are correlated with baseline serum HBsAg and HBeAg levels and might predict a shorter interval to HBeAg loss and seroconversion during antiviral treatment. *Infect Genet Evol* 2015;33:261-8. doi: 10.1016/j.meegid.2015.05.013 [published Online First: 2015/05/16]
